# *Passiflora agudeloi* (Passifloraceae, subgenus *Decaloba*): A new endemic species from cloud forests in the Central Cordillera of the Colombian Andes

**DOI:** 10.3897/phytokeys.271.175046

**Published:** 2026-03-13

**Authors:** Larri Álvarez Rodas, John Ocampo, Santiago Montoya-Rubio

**Affiliations:** 1 Universidad del Quindío, Doctorado en Ciencias – Biología, Cra. 15 #12N, Armenia, Quindío, Colombia Grupo de Investigación en Biodiversidad y Biotecnología de la Universidad del Quindío (GIBUQ) Armenia Colombia; 2 Grupo de Investigación en Biodiversidad y Biotecnología de la Universidad del Quindío (GIBUQ), Cra. 15 #12N, Armenia, Quindío, Colombia Universidad del Quindío Armenia Colombia; 3 Universidad Nacional de Colombia Sede Palmira, Facultad de Ciencias Agropecuarias, Cra. 32 Chapinero, vía a Candelaria, Palmira, Valle del Cauca, Colombia Grupo de Estudio en Botánica de la Universidad del Quindío (GEBUQ) Armenia Colombia; 4 Grupo de Estudio en Botánica de la Universidad del Quindío (GEBUQ), Cra. 15 #12N, Armenia, Quindío, Colombia Universidad Nacional de Colombia Sede Palmira Palmira Colombia

**Keywords:** Endemism, new taxon, passionflower, Quindío, Tolima

## Abstract

*Passiflora agudeloi* is herein recognised as a distinct species, based on a unique combination of vegetative, floral and seed characters. It differs from morphologically related species with smaller vegetative and floral structures, such as *P. hyacinthiflora* and *P. bicuspidata*, by its larger leaves and flowers. Unlike *P. trinervia*, which exhibits a hypanthium exceeding 10 cm in length, *P. agudeloi* possesses a shorter hypanthium (0.4–0.5 cm). It can be separated from *P. sierrae* by the absence of foliar nectaries and by its longer petioles and non-pendent flowers. The new species is clearly distinct from closely-related species *P. azeroana* in many key characters, including flower size and colouration, length and pigmentation of petioles, peduncles and stipules, shape and distribution of laminar glands, as well as seed morphology and ornamentation. Its preliminary conservation assessment, categorised as Critically Endangered (CR) under the IUCN Criteria, underscores the urgent need to implement both in situ and ex situ conservation strategies to ensure its long-term survival.

## Introduction

The Andes Mountain range is recognised as one of the most biologically diverse regions in the world, harbouring an exceptional richness of plants, fish, mammals, insects, birds and other organismal groups (Myers et al. 2000; Hazzi et al. 2018). The flora of the Andean region includes about 28,691 vascular plant species and Colombia accounts for 38% of this biological richness (Pérez-Escobar et al. 2022). The Department of Tolima, located in the central Colombian Andes, constitutes a region of exceptional biological importance. More than 3,000 plant species have been documented within its territory, reflecting high floristic richness across an extensive elevational gradient extending from below 400 m to above 5,000 m a.s.l. (IGAC 2018).

The genus *Passiflora* L. is the most species-rich in the family Passifloraceae Juss. ex Roussel, with around 620 species and is distributed mainly in America (Boza et al. 2018). *Passiflora* is a genus primarily represented by herbaceous or woody, tendril-climbing vines, although a few species occur as trees or shrubs. The genus is well known for its complex, radially symmetrical flowers, alternate leaves with stipules and the presence of petiolar and/or laminar nectary glands (Killip 1938; Ulmer and MacDougal 2004). Fruits are generally fleshy berries, occasionally capsules, bearing numerous seeds embedded in a pulpy matrix and show striking interspecific variation in size, shape and colouration (Ocampo and Coppens d’Eeckenbrugge 2017).

About 220 species of this family are reported from Colombia, grouped into five genera, of which *Passiflora* has approximately 190 species. Colombia is the country with the largest number of native passionflower species in the world (Ocampo et al. 2007, 2010, 2021). This number is continually growing across the country, with new studies utilising fieldwork and herbarium specimens. A good number of species new to science have been discovered in Colombia in the last ten years, such as *P. munchiquensis* A. Hernández, *P. gustaviana* Ocampo & Molinari, *P. quimbayensis* Ocampo & Forero, *P. kumandayi* M.A. Buitrago A. & Coca, *P. nebulosae* J. Restrepo & Ocampo, *P. mistratensis* Kuethe & Vanderplank and *P. dulimae* Ocampo, Lozano-Cif., Villanueva & Corrales-Bravo. The species of *Passiflora* play an important role in ecosystems through their multiple interactions with other organisms, especially pollinators and are considered indicator species of biodiversity (Ocampo et al. 2010). Despite their richness and diversity, they are in constant danger of extinction, with nearly 110 species listed under some stage of threat to their populations (Ocampo et al. 2010).

*Passiflora* subg. *Decaloba* (DC.) Rchb. is one of the six subgenera of *Passiflora* currently recognised according to morphological, cytological and phylogenetic-genomic evidence (Muschner et al. 2012; Krosnick et al. 2013; Ocampo and Coppens d’Eeckenbrugge 2017; Sader et al. 2019; Cauz-Santos et al. 2020). *Passiflora* subg. *Decaloba* includes relatively small climbers and vines with the presence of petiolar and/or laminar nectary glands and small flowers and fruits (Killip 1938; Milward-de-Azevedo et al. 2012; Krosnick et al. 2013). It is the second-largest group, with approximately 260 species, followed by *P.* subg. *Passiflora*, which includes more than 280 species (Boza et al. 2018). *Passiflora* subg. *Decaloba* is divided into seven supersections and five sections (Feuillet and MacDougal 2003), distributed in America, with a small group of 24 species found in Southeast Asia and Oceania. Species of *P.* sect. *Decaloba*, characterised by laminar nectaries, corona filaments in one or two series and fruit berries (Feuillet and MacDougal 2003), occur in the Neotropics and have their centre of diversity in the northern Andes (Hernández 2003; Acha et al. 2021). Within *P.* sect. *Decaloba*, five species exhibit distinctive morphological traits, including rose-purple or purple or violet-coloured flowers and a uniseriate corona. These species occur in the Colombian Andes (*P. azeroana* L. Uribe, *P. bicuspidata* (H. Karst.) Mast., *P. hyacinthiflora* Planch. & Lin and *P. trinervia* (Juss.) Poir.) and in the Sierra Nevada de Santa Marta (*P. sierrae* L.K. Escobar), at elevations above 2,500 m a.s.l. *Passiflora bicuspidata*, *P. hyacinthiflora* and *P. trinervia* are characterised by pendulous flowers with a hypanthium length exceeding 3 cm, whereas *P. sierrae* and *P. azeroana* (with semi-erect flowers) bear short flowers with a hypanthium length of less than 0.8 cm.

The Central Cordillera of the Colombian Andes constitutes an area of remarkable floristic diversity and endemism, with particular presence in the transitional zone between the Departments of Caldas, Quindío, Risaralda and Tolima. Nevertheless, this region and others across the country are expected to undergo drastic changes over the years due to anthropogenic activities, such as livestock farming, forest plantations, deforestation and gold mining (Tejedor et al. 2012). In this area, floristic inventories have reported several species of *Passiflora* growing in fragmented forests over 2,500 m a.s.l., some of these belonging to *P.* sect. *Decaloba*, such as *P. alnifolia* Kunth, *P. apoda* Harms, *P. chelidonea* Mast., *P. kumandayi* M.A. Buitrago A. & Coca, *P. mollis* Kunth and *P. trinervia* (Juss.) Poir. (Hernández 2003; Ocampo et al. 2007, 2010). Despite substantial floristic research efforts, extensive areas with suitable habitats either remain botanically unexplored or have only been partially investigated in this region.

In this context, the present study describes *Passiflora agudeloi*, a new species of *P.* subg. *Decaloba* sect. *Decaloba*, occurring in the Andean region of the Municipality of Cajamarca, Department of Tolima, Colombia. Its diagnostic characters are illustrated and compared with those of morphologically similar taxa, including *P. azeroana*, *P. bicuspidata*, *P. hyacinthiflora*, *P. sierrae* and *P. trinervia*.

The description is supported by detailed ecological and morphological evidence derived from both living plants observed in the field and specimens preserved in herbaria. This taxon is morphologically distinct within *P.* sect. *Decaloba* due to a unique combination of vegetative and floral characters and is restricted to high-elevation habitats on the central-eastern flank of the Central Cordillera.

## Materials and methods

Environmental studies conducted by the universities of Quindío and Caldas in Colombia, as part of the Environmental Monitoring Research Programme, were carried out between 2012 and 2015 in the La Luisa area of the Municipality of Cajamarca (Tolima Department). The study area is located on the eastern slope of the Central Cordillera of the Colombian Andes, bordering the Department of Quindío, at elevations ranging from 2300 to 3200 m a.s.l. This elevation corresponds to the high Andean zone, characterised by low temperatures (˂15 °C), high relative humidity (˃80%) and marked diurnal thermal variations.

Eleven specimens of *Passiflora* obtained during these expeditions were photographed, processed and subsequently deposited in the herbaria of the University of Quindío (**HUQ**) and the University of Caldas (**FAUC**). Amongst the collected material, two individuals growing 1.5 metres apart were studied and initially identified as *P. aff. azeroana* L. Uribe and belonging to *P.* subg. *Decaloba* sect. *Decaloba*. Due to the original doubt, these specimens were re-examined 13 years later with a detailed comparison with the protologues of *P. azeroana* (Uribe 1955) and *P. sierrae* (Escobar 1989) and with the application of taxonomic keys to *Decaloba* (Killip 1938; Hernández 2003; Ulmer and MacDougal 2004).

To corroborate this hypothesis of taxonomic affinity and to determine whether this putative new species is distributed in other regions of Colombia or outside the country, specimens of *P.* subg. *Decaloba* sect. *Decaloba* were examined in the major Colombian herbaria (COL, CDMB, CUVC, FAUC, HECASA, HUA, HUQ, JAUM, JBB, MEDEL, SURCO, TOLI, TULV, UDBC, UIS, UTMC and VALLE), as well as in herbarium collections in other countries (K, MA, MPU, MOL, UCR, P and USM). In addition, high-resolution images of type collections from herbaria, such as F, GH, QCA, MO, NY, TEX and US, available through JSTOR Plants Science (2025), were reviewed. Georeferenced images of botanical observations from iNaturalist (https://www.inaturalist.org) were also examined. Main taxonomic monographs and reference works on *Passiflora* (Killip 1938; Uribe 1955; Hernández 2003; Ulmer and MacDougal 2004) were also consulted to evaluate the morphological affinities and to clarify the taxonomic placement of the specimen.

A total of 25 vegetative and reproductive morphological descriptors, selected by Ocampo and Coppens d’Eeckenbrugge (2017) for *P.* subg. *Decaloba*, were used to describe and compare the putative new species with related taxa. These descriptors are primarily associated with the stipule, leaf, flower and seed. The morphological characterisation of the new species was conducted in situ using fresh material from two living individuals collected in the field trip. In the case of related taxa, morphological data were obtained from the research of Ocampo and Coppens d’Eeckenbrugge (2017) and additional collections from at least two living individuals of each species studied (*P. azeroana*, *P. bicuspidata*, *P. hyacinthiflora*, *P. sierrae* and *P. trinervia*). To encompass the maximum variation, the dataset was complemented with measurements obtained from herbarium specimens examined and from in situ photographs of living individuals observed on iNaturalist. Five measurements for each quantitative vegetative, floral and seed descriptor were taken from each individual plant. The terminology used for the descriptions was based on Killip (1938), Puri (1947, 1948), Tillett (1988) and Ulmer and MacDougal (2004).

Personal field observations, herbarium and documented observations from iNaturalist were databased (see Suppl. material 1) to generate a dot distribution map of the occurrence of the new species and its closest relatives using QGIS v.3.36. In addition, a preliminary conservation status for the new species was determined according to IUCN Categories and Criteria in IUCN (2024), based on the calculation of the extent of occurrence (**EOO**) and area of occupancy (**AOO**) using GeoCAT (Bachman et al. 2011) with the default setting of 2 km^2^ grid.

## Results

Taxonomic keys, based on morphological comparisons, indicated that the specimens, initially considered closely related to *P. azeroana*, represent a new species. In addition, 15 qualitative and quantitative morphological characters were identified that highlight the differences between this new species and its closest relative, *P. azeroana* (Table 1).

**Table 1.** Summary and integrative comparisons of the main morphological characters distinguishing *P. agudeloi* from its closest relative species, *P. azeroana*.

**Taxon / Character**	***P. agudeloi***	***P. azeroana***
Distribution	Central-eastern flank of Central Cordillera 3,200 m a.s.l.	North-western flank of Eastern Cordillera / South–eastern flank of Central Cordillera 2,500–3,091 m a.s.l.
Stipules	2–4 × 1 mm, greenish-reddish purple	5–10 × 2–3 mm, reddish-purple
Leaves	Ovoid-oblong	Oblong
Angle between lateral lobes	70–75°	60–70°
Laminar glands	Two symmetrical series of clear nectary; 3–15; in the middle of the lateral veins	Two pairs of conspicuous ocelli; the first pair at the base and the second pair in the middle of the lamina, pronounced; 5–7; in the middle of lateral veins
Flowers	4.6–4.8 cm diam.	7.6–8 cm diam.
Outer corona filaments	Curved at the apex, light green at the base, whitish in the centre and yellowish apically	Straight filaments, purple at the base and the middle and greyish-purple apically
Operculum	Reddish-purple	Purple
Sepals	Linear oblong, 2.5–3 × 0.4–0.5 cm, apex obtuse-acute, purple-violet	Linear oblong, 3.8–4 × 0.4–0.5 cm, apex obtuse-acute, magenta
Petals	Linear oblong, 1.2–1.5 × 0.3 cm, apex acuminate, dark fuchsia at edge and light in centre	Linear oblong, 2–2.5 × 0.3–0.4 cm, apex acute, magenta
Ovary	Ovoid-oval, puberulent	Ovoid-rounded, hirsute
Seed	Subspherical, 0.42–0.48 × 0.34–0.38 cm	Ovoid-ellipsoidal, 0.37–0.4 × 0.24–0.3 cm
Seed ornamentation	Rugose-reticulate ornamentation with radial grooves and prominent ridges in radial pattern	Reticulate-rugose ornamentation with irregular ridges and deep folds

### Taxonomic treatment

#### Passiflora agudeloi

Taxon classificationPlantaeMalpighialesPassifloraceae

L.A.Rodas, Ocampo & S.Montoya-R sp. nov.

87D1CE0B-34EB-55A0-BE6B-551709F804F8

urn:lsid:ipni.org:names:77377660-1

Figs 2, 3

##### Type.

**Colombia** • Tolima: Cajamarca, Vereda La Luisa, límites con Quindío, 4.471556, -75.494111, 3200 m a.s.l., 04 Jul 2012 (fl, fr). *L. Álvarez, J.M. Duque, J. Baquero & L. Cifuentes 087* (holotype: HUQ!; isotype: FAUC!).

##### Diagnosis.

*Passiflora agudeloi* is distinguished from its relative’s species by the combination of leaves wider than 3 cm, petioles longer than 1.5 cm and lacking foliar nectaries and semi-erect flowers 4.6–4.8 cm in diameter. Unlike *P. trinervia*, which exhibits a hypanthium exceeding 10 cm in length, *P. agudeloi* possesses a hypanthium shorter than this threshold. The new species differs from *P. hyacinthiflora* and *P. bicuspidata* in its larger leaves and flowers and from *P. sierrae* by its longer petioles, absence of nectaries and non-pendent flowers. The species is most similar to *P. azeroana*, but differs by having smaller flowers 4.6–4.8 cm in diam. (vs. 7.6–8 cm), shorter sepals 2.5–3 cm long (vs. 3.8–4 cm), narrower petals 1.2–1.5 cm long (vs. 2–2.5 cm), outer corona filaments curved at the apex, light green at the base, whitish at the centre and yellowish at the tip (vs. purple at base and middle, greyish-purple at apex), a puberulent ovoid-oval ovary (vs. ovoid-rounded, hirsute ovary) (Fig. 4), and seeds subspherical 0.42–0.48 × 0.34–0.38 cm (vs. ovoid-ellipsoidal, 0.37–0.4 × 0.24–0.3 cm), rugose-reticulate ornamentation with radial grooves and prominent ridges in radial pattern (vs. reticulate-rugose with irregular ridges and deep folds).

##### Description.

Liana up to 4 m in length. ***Stem*** subterete, cylindrical, striate and lustrous, puberulent, green to purple in colour. Internode length 6.3–7.6 cm. ***Tendrils*** thick, markedly lignified at the base, thinner towards the apex, glabrous and shiny, wine-coloured, exceeding 15 cm in length. ***Stipules*** setaceous, acute apex, glabrous, purple to wine-coloured, 0.2–0.4 × 0.1 cm. ***Petioles*** 1.9–2.7 cm long, pubescent and striate on the adaxial surface, wine-coloured, eglandular. ***Leaves*** 3-lobed, with lateral lobes larger than the central one, 70–75° between lateral lobes, 8–10 × 4.5–6.3 cm, oblong, glossy, adaxially glabrous, abaxially pulverulent along the central veins; lateral lobes with acute apices, protruding between 0.3 and 1 cm beyond the central lobe, separated from each other by 2.0–2.7 cm; blade base cordate, margin entire, subcoriaceous; adaxially dark green, abaxially light green with three conspicuous wine-coloured veins; unequal width between the central and lateral veins, ranging from 0.9 to 1.5 cm; two extrafloral nectaries present at the base of the blade and 3 to 15 additional extrafloral nectaries distributed along the central and lateral veins. ***Peduncles*** paired or solitary, 2.9–3.3 cm long, cylindrical, lustrous and glabrous, dark wine-coloured. ***Pedicels*** of the same colour, 0.62–0.69 cm long, thickened, cylindrical, glabrous. ***Bracts*** 0.2–0.4 cm long, linear to setaceous, subopposite, dark purple. ***Flowers*** 3.5 × 4.7 cm in diameter, semi-erect, lustrous, glabrous, reddish-purple to purple. ***Hypanthium*** 0.4–0.5 (to the base of the sepal) × 0.7–0.8 cm, depressed-globose, with two protrusions beneath each sepal on the outer surface. ***Sepals*** 2.5–3 × 0.4–0.5 cm, linear-lanceolate, broader at the base, canaliculate, dark violet along the margins, paler in the centre, with three prominent veins, apex obtuse to acute, membranous. ***Petals*** markedly 1.2–1.5 × 0.3 cm, shorter than sepals, linear-lanceolate, broader at the base, canaliculate, with dark violet margins, fuchsia-pink centre and apex, membranous, with multiple veins, apex acute to acuminate. ***Corona*** of filaments in a single series (radii), 0.47–0.5 cm long, green at the base and bright yellow at the apex, curved towards the androgynophore. ***Androgynophore*** 2.8–3 × 0.1–0.2 cm, dark violet, glabrous, measuring 1.2 cm from the base of the ovary to the apex of the stigmas. ***Gynophore*** with styles 0.6 cm long, dark olive green. ***Stigmas*** 0.1–0.2 × 0.1–0.2 cm, capitate, light lemon green. ***Filament*** 0.75–0.77 cm long, dark wine-coloured with a greenish apex. ***Anthers*** 0.4–0.5 × 0.1–0.2 cm, yellow on the adaxial surface, violet-grey on the abaxial surface. ***Ovary*** 0.44–0.45 × 0.13–0.15 cm, ovoid to oval, puberulent, light lemon green. ***Fruit*** 1.8 × 1.5 cm, ovoid, puberulent, light green, darkening when ripe. ***Seed*** 0.42–0.48 × 0.34–0.38 cm, sub–spherical, dark brown, dentate margin, rugose–reticulate ornamentation with radial grooves and prominent ridges in radial pattern, a prominent central horn at the apex and an acute base.

##### Phenology.

This plant was observed with flowers in July–August and September–October and with fruits in August and October–November.

##### Etymology.

The species is named in honour of Colombian botanist Carlos Alberto Agudelo Henao, whose academic career has been dedicated to science education, conservation and botany. Throughout his career as professor, he has fostered interest in these fundamental topics, educating new generations with the capacity to care for and preserve biodiversity and ecosystem health. Furthermore, his vision and contributions have been key to enriching the collection of the Herbarium of the University of Quindío (HUQ) for more than 40 years. He has also played a fundamental role in the creation and consolidation of undergraduate and graduate programmes in biological sciences, strengthening academic and scientific training in the country.

##### Distribution and ecology.

**Figure 1.**
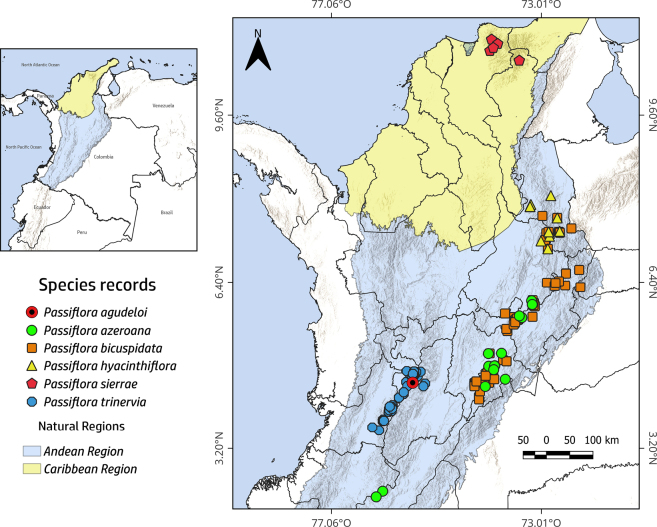
Map of the geographical distribution of *Passiflora agudeloi* L.A.Rodas, Ocampo & S.Montoya-R (red circles), compared to their closest relatives *P. azeroana* (green circles) and allied species, *P. bicuspidata* (orange squares), *P. hyacinthiflora* (yellow triangles), *P. sierrae* (red pentagons) and *P. trinervia* (blue octagons), framed within the Colombian Andean and Caribbean regions.

This is a rare, endemic species from high Andean cloud forests of the eastern slopes of the Central Cordillera of the Colombian Andes. Its distribution is restricted to the Municipality of Cajamarca, in the Department of Tolima, along the border with Quindío, at an elevation of 3,200 m a.s.l. (Fig. 1). The new species is a climbing plant growing on trees and shrubs at the edge of secondary cloud forests with humid soils. The climatic conditions in this area exhibit a range of temperatures of 5.1–13.8 °C and an annual mean rainfall of about 1736 mm (IDEAM 2025).

**Figure 2.**
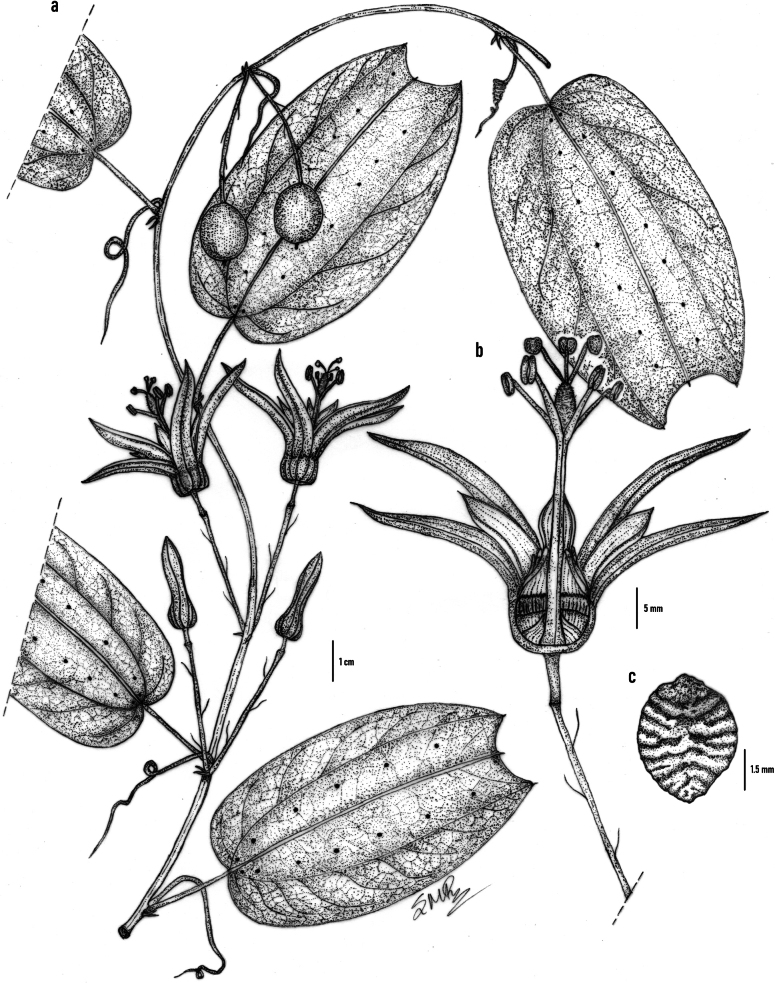
*Passiflora agudeloi* L.A.Rodas, Ocampo & S.Montoya-R. **a**. Detail of a flowering branch; **b**. Flower in traversal section; **c**. Seed. Drawn by Santiago Montoya-R from the holotype (*L. Álvarez, J.M. Duque, J. Baquero & L. Cifuentes 087*; HUQ!).

##### Conservation status.

*Passiflora agudeloi* presents a high degree of ecological specialisation; this, in turn, could make the species particularly vulnerable to environmental change and habitat fragmentation. The new species occurs outside the borders of any Colombian protected areas (e.g. Parque Nacional Natural Los Nevados) and is currently known from two individuals (Fig. 1). According to the IUCN Red List Criteria and thresholds B and D (2024), the species qualifies for the category Critically Endangered (CR). This assessment is based on its extremely restricted geographic range (criterion B), as indicated by a single locality outside protected areas and on its extremely small population size (criterion D), given that only one individual has been recorded to date. Such conditions imply a very high risk of extinction and highlight the urgent need for both *in situ* and *ex situ* conservation measures.

**Figure 3.**
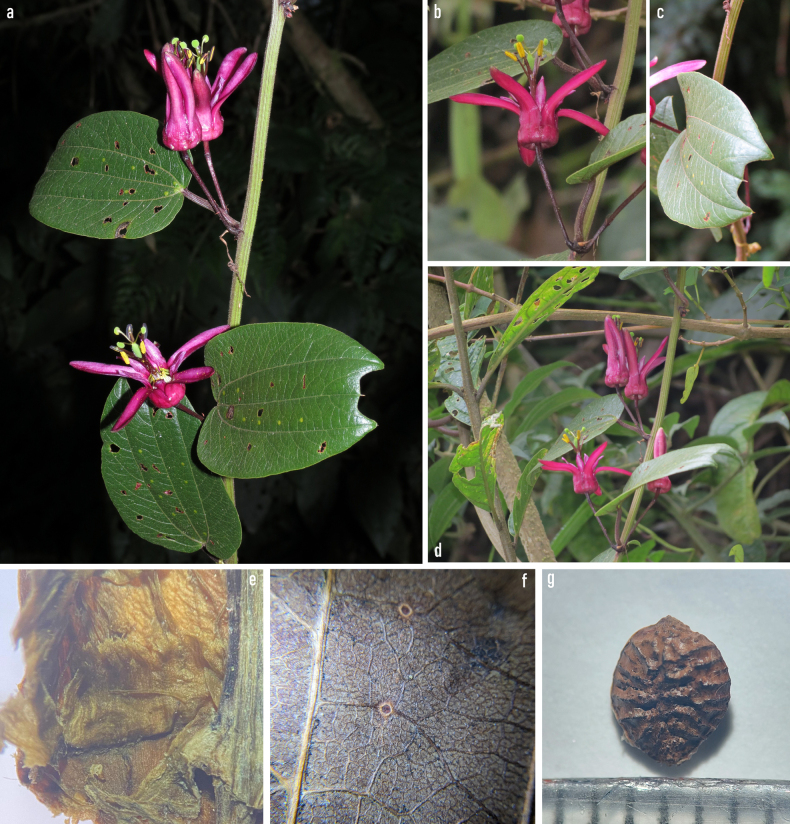
Vegetative and reproductive structures of *Passiflora agudeloi* L.A.Rodas, Ocampo & S.Montoya-R. **a**. Habit and basic characteristics of the plant, showing the striated stem, three-lobed leaves with lateral veins larger than the central vein when mature, petioles, peduncles and pedicels with the characteristic wine-red colour of the new species. Flowers in pairs of striking dark fuchsia colour; **b**. Fully erect and mature flower; **c**. Mucronate apex; **d**. Flower maturation; **e**. Limen and operculum in the traversal section; **f**. Laminar nectaries; **g**. Seed ornamentation.

**Figure 4.**
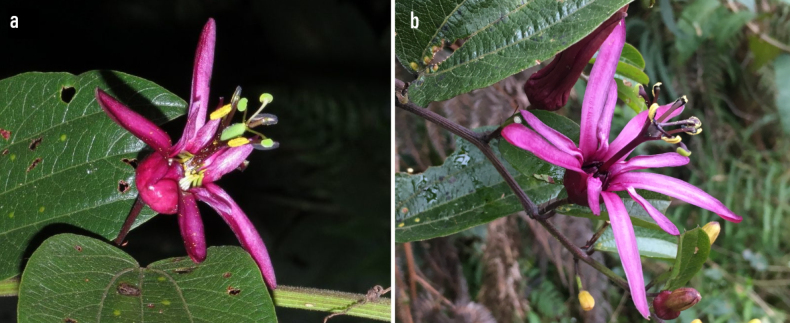
Comparative floral structure of (**a**) *Passiflora agudeloi* (Tolima, eastern slope of the central Andes Mountain range) and (**b**) *Passiflora azeroana* (Cundinamarca, western slope of the northern eastern Andes Mountain range). Photos by Larri Alvarez (**a**) and John Ocampo (**b**).

##### Additional specimens examined

**(paratypes). Colombia** • **Tolima**: Cajamarca, Vereda la Luisa, límites con Quindío, 4.471556°N, 75.494111°W, 3200 m a.s.l., 06 Sep 2012 (fl), *A.F. Bohórquez-O, L. Álvarez, J.M. Duque, J. Baquero & L. Cifuentes 463* (FAUC!).

##### Selected specimens examined

**(closest relatives)**. *Passiflora azeroana*. **Colombia** • **Boyacá**: Arcabuco, Finca “Ortigales”, 2850 m, 5.716667°N, 73.383334°W, 03 Jan 1970, *G. Huertas & L. Camargo 6833* (COL); • Duitama, Corregimiento de Virolín, Finca “La Sierra”, 2500–2600 m a.s.l., 6.040201°N, 73.194829°W, 1976, *G. Lozano et al. 2527* (COL); • Arcabuco, La Cumbre, 2300 m a.s.l., 5.75°N, 73.433334°W, 07 Oct 1976, *G. Lozano & J. Díaz 3718* (COL, UTPC). • **Cundinamarca**: Bogotá, Torca, 2800–3000 m a.s.l., 4.784444°N, 74.023889°W, 19 Oct 2003, *R. Díaz & M. Ardilla 011 MA-RD* (COL); • Chapinero, Cerro Usaquén, 2970 m a.s.l., 18 Jul 2010, *G. Morales 3187* (JBB); • Chipaque, Calderitas, 4.394722°N, 74.090416°W, 3031 m a.s.l., 25 Oct 2013, *G. Morales 3649* (JBB); • Fomeque, 2900 m a.s.l., 4.533333°N, 73.703889°W, 22 Mar 1969, *G. Huertas & L. Camargo 6745* (COL). • **Huila**: La Plata, Vereda Arrabal, 2380 m a.s.l., 2.373152°N, 76.073071°W, 10 Jan 1984, *G. Lozano et al. 4338* (HUA!); • Isnos, Plan de Achupayal de Perico, 2700 m a.s.l., 17 Jan1973, *C.E. Acosta 16* (COL); • La Plata, Páramo de la Candelaria, Inspección de Santa Leticia, 2370 m a.s.l., 14 Jul 1971, *Díaz-Piedrahita et al. 602* (COL). • **Santander**: Charalá, Virolín, 2500 m a.s.l., 14 May 1976, *G. Lozano 2527* (COL); • Charalá, Virolín, 2500 m a.s.l., Aug 1963, *D. Goitia s.n*. (UDBC). *Passiflora bicuspidata*. **Colombia** • **Arauca**: Tame-Sácama, 2000 m a.s.l., 3 Jun 1995, *R. Manrique & R. Castillo* 106 (UPTC). **Boyacá** • Socha, 2580 m a.s.l., 2 Nov 1987, *C. Orozco et al. 2016* (US!). • **Cundinamarca**: La Calera, El Salitre, 2990 m a.s.l., 20 Jun 2003, *G. Morales et al. 2137* (JBB); • Sibaté, Carretera entre Sibaté y Fusagasuga, 2600 m a.s.l., 3 Feb 1883, *Lehmann 2498* (K!). • **Norte de Santander**: Pamplona, 2640 m a.s.l., 6 Aug 2016, *L.R. Sánchez et al. 16935* (HECASA); • Cucutilla, 3500 m a.s.l., 26 May 2012, *L.R. Sánchez & A. Ojeda 14363* (HECASA); • Pamplona, 2950 m a.s.l., 20 Jul 2012, *C.B. Rojas & L.R. Sánchez 106* (HECASA); • Pamplona, 2700 m a.s.l., 26 Nov 1999, *L.R. Sánchez & P. Montaño 4461* (HECASA); • Pamplona, 3100 m a.s.l., 26 Jul 2002, *L.R. Sánchez & A. Castellanos 6901* (HECASA). • **Santander**: Concepción, 3280 m a.s.l., 24 Jul 2014, *L.R. Sánchez & C.B. Rojas 16137* (HECASA); • Vetas, 3500 m a.s.l., 16 Nov 2002, *J.A. Mejía & J.A. Hernández 170* (CDMB); • Tona, 3400 m a.s.l., 20 Feb 2002, *J. Tejada et al. 36* (UIS). *Passiflora hyacinthiflora*. **Colombia** • **Boyacá**: Duitama, 3065 m a.s.l., 3 Nov 2005, *L. Rosero 217* (UTPC). • **Norte de Santander**: Abrego, 3400 m a.s.l., 13 Jan 2002, *L.R. Sánchez & D. Pérez 6193* (HECASA); • Pamplona, Páramo de las Cruces, 2700 m a.s.l., 1 Dec 1846, *N. Funck & L.J. Schlim 1383* (G!, MPU!). *Passiflora sierrae*. **Colombia** • **Magdalena**: Sierra Nevada de Santa Marta, 3070–3100 m a.s.l., 9 Oct 1959, *J. Cuatrecasas & R. Romero-Castañeda 24675* (US!); • Sierra Nevada de Santa Marta, 3070–3100 m a.s.l., 9 Oct 1959, *J. Cuatrecasas & R. Romero-Castañeda 24675* (COL!); • Ciénaga, Laguna Chubdala, 3800 m a.s.l., 24 Feb 1993, *Eduino Carbonó 3110* (UTMC). *Passiflora trinervia*. **Colombia** • **Quindío**: Salento, Valle de Cocora, 2900 m a.s.l., 4.63763°N, -75.4892°W, May 1989, *L.M. Álvarez-Mejía 926* (FAUC); • Calarcá, Vereda Planadas, 2800 m a.s.l., 6 Sept 1993, *M.C. Vélez et al. 3412* (HUQ). • **Valle del Cauca**: Bugalagrande, Cuchilla de Barragán, 3320 m a.s.l., 20 Mar 1976, *J. Cuatrecasas 20241* (VALLE); • Tuluá, Corredor Barragán, 2800 m a.s.l., 24 Jun 1983, *W. Devia 192* (TULV); Corredor Santa Lucía, 2900 m a.s.l., 3 Nov 1987, *W. Devia & F. Prado 1981* (TULV).

### Taxonomic key to *Passiflora agudeloi* and related species

**Table:** 

1	Hypanthium longer than 10 cm	***P. trinervia***
–	Hypanthium shorter than 10 cm	**2**
2	Leaves up to 2.5 cm wide; flowers less than 0.8 cm in diameter	**3**
–	Leaves wider than 3 cm; flowers 3.5 cm in diameter or larger	**4**
3	Stem flattened and striate; calyx base acute; ovary hirsute/tomentose	***P. hyacinthiflora***
–	Stem angular, longitudinally sulcate; calyx base globose; ovary glabrous	***P. bicuspidata***
4	Petioles shorter than 1 cm, with 1 pair of nectaries at the apex; leaf with lateral lobes reduced relative to the central lobe; flowers pendent, up to 4 cm in diameter	***P. sierrae***
–	Petioles longer than 1.5 cm, devoid of nectaries; leaf apex bilobed; flowers semi- erect, more than 4.5 cm in diameter	**5**
5	Flowers 7.6–8 cm in diameter; sepals 3.8–4 cm long; petals 2–2.5 cm long; ovary hirsute; outer corona filaments purple at base and middle, apically greyish-purple. Seed ovoid-ellipsoidal, reticulate	***P. azeroana***
–	Flowers 4.6–4.8 cm in diameter; sepals 2.5–3 cm long; petals 1.2–1.5 cm long; ovary puberulent; outer corona filaments light green at base, whitish in centre, apically yellowish. Seed subspherical, rugose	***P. agudeloi***

## Discussion

The description of *Passiflora agudeloi* as a new species within *P.* subg. *Decaloba* reinforces the importance of the Colombian Andes, particularly the region between Tolima and Quindío, as a hotspot for plant diversity and endemism. As previously reported, this region exhibits exceptional floristic richness due to its complex topography, variety of microhabitats and pronounced altitudinal gradients (Pérez-Escobar et al. 2022). Nevertheless, this richness remains largely under-documented, partly due to the limited access and the ongoing transformation of Andean ecosystems.

The morphological comparison and resulting dichotomous key clearly demonstrate that *P. agudeloi* is distinguishable from its relative species (*P. azeroana*, *P. bicuspidata*, *P. hyacinthiflora*, *P. sierrae* and *P. trinervia*). A notable morphological trait shared by species related to *P. agudeloi* is the markedly short hypanthium, measuring less than 0.8 cm in length. This character is also observed in *P. azeroana* and *P. sierrae*. However, *P. sierrae* differs from the former taxa mainly by the presence of pendulous flowers and a single pair of nectaries located at the apex of the petiole. Owing to the presence of petiolar glands, *P. sierrae* should be excluded from *P.* sect. *Decaloba*, as the absence of petiolar glands constitutes a defining diagnostic feature of this section. For these reasons and based on a consistent suite of vegetative and reproductive characters (Table 1), *P. azeroana* is the closest species to *P. agudeloi*. Amongst the most conspicuous differences are the smaller flower size (4.6–4.8 cm vs. 7.6–8 cm), the shorter sepals and petals and the puberulent ovoid-oval ovary in *P. agudeloi*, as opposed to the hirsute ovoid-rounded ovary in *P. azeroana*. These floral traits are of taxonomic importance within *Passiflora* and have been used successfully in previous studies to delimit species within *P.* subg. *Decaloba* (Ocampo and Coppens d’Eeckenbrugge 2017). The differences in outer corona filament colour and structure also provide key diagnostic features. While both species lack inner corona filaments, the outer filaments of *P. agudeloi* are light green at the base, whitish in the middle and yellowish at the apex, with a distinct apical curvature, contrasting with the purplish and straight filaments of *P. azeroana*. This character is particularly relevant because corona morphology is often linked to pollinator specificity and may reflect ecological divergence between the species (Ulmer and MacDougal 2004). The stipule size and colour, as well as laminar gland arrangement, further support the separation of *P. agudeloi* as a distinct taxon. In *P. agudeloi*, stipules are smaller (2–4 mm vs. 5–10 mm) and show a greenish-reddish purple hue and the laminar glands are more numerous (3–15) and aligned in symmetrical series, as opposed to the more restricted and paired arrangement in *P. azeroana*. Even more striking are the differences observed in the size, shape (subspherical, 0.42–0.48 × 0.34–0.38 cm vs. ovoid-ellipsoidal, 0.37–0.4 × 0.24–0.3 cm) and ornamentation of the seeds (Table 1), which provide additional diagnostic characters that further delimit *P. agudeloi* from *P. azeroana*.

These morphological distinctions are complemented by ecological and biogeographic data. *Passiflora agudeloi* is an endemic species restricted to the central-eastern flank of the Central Cordillera of the Colombian Andes, occurring at an elevation of 3,200 m a.s.l. In contrast, *P. azeroana* exhibits a broader distribution, inhabiting the north-western flank of the Eastern Cordillera and the south-eastern flank of the Central Cordillera, within an elevational range of 2,500–3,091 m a.s.l. The observed spatial disjunction, combined with morphological divergence, supports the hypothesis of allopatric speciation within *P.* sect. *Decaloba* in montane environments. The occurrence of *P. agudeloi* in a single orographic unit at a high elevation suggests marked ecological specialisation, potentially associated with transitional zones between montane forest and paramo ecosystems. These findings are particularly relevant for conservation of the new species. The narrow distribution of *P. agudeloi* renders it particularly vulnerable to habitat disturbance and the impacts of climate change, warranting its classification as Critically Endangered (CR). Conversely, *P. azeroana*, with its wider geographical and elevational range, may exhibit comparatively greater resilience to environmental pressures.

Taxonomic work in the genus *Passiflora*, particularly within *P.* subg. *Decaloba*, has been challenged by a high morphological variability and convergent evolution amongst its species (Hernández 2003; Ocampo and Coppens d’Eeckenbrugge 2017; Acha et al. 2021). Nonetheless, the use of updated taxonomic keys and careful comparative morphological analysis proves effective in refining species boundaries, as demonstrated in this study. Moreover, the combination of long-term fieldwork and comprehensive herbarium specimen review remains an effective strategy for identifying new taxa (Heberling 2022).

This discovery also emphasises the need to continue systematic floristic inventories and environmental monitoring programmes in poorly explored areas. Strengthening collaborations between academic institutions, such as the Universities of Quindío and Caldas can be instrumental in supporting biodiversity documentation and protection. Ultimately, the description of *P. agudeloi* enhances the systematic understanding of *P.* subg. *Decaloba*, while simultaneously contributing critical baseline data for the development of regionally informed conservation strategies and the sustainable management of high Andean ecosystems. The recognition of *P. agudeloi* contributes to our understanding of the remarkable diversification of *Passiflora* in Andean montane ecosystems and highlights the importance of continued fieldwork and herbarium studies for documenting plant diversity in Colombia. Fieldwork and integrative taxonomic approaches are essential to document the diversity of *Passiflora* in Colombia, a country that remains the global centre of diversity for the genus.

## Supplementary Material

XML Treatment for Passiflora agudeloi

## Additional information

### Use of AI

No use of AI was reported.

### Author ORCIDs

Larri Álvarez Rodas https://orcid.org/0000-0003-0700-7371

John Ocampo https://orcid.org/0000-0002-2720-7824

Santiago Montoya-Rubio https://orcid.org/0009-0000-2958-6154

### Data availability

All of the data that support the findings of this study are available in the main text or Supplementary Information.

## Supplementary materials

Supplementary material 1 Personal field observations, herbarium and documented observations from iNaturalist were databased Data type xlsx https://binary.pensoft.net/file/1561050This dataset is made available under the Open Database License (http://opendatacommons.org/licenses/odbl/1.0/). The Open Database License (ODbL) is a license agreement intended to allow users to freely share, modify, and use this Dataset while maintaining this same freedom for others, provided that the original source and author(s) are credited. Larri Álvarez Rodas, John Ocampo, Santiago Montoya-Rubio
